# Resilience, Mental Toughness, and Physical Activity Levels: A Survey of Doctors From Different Generations and Specialities

**DOI:** 10.7759/cureus.101834

**Published:** 2026-01-19

**Authors:** Ryan S Ting, Siew-Piau Tan, Michael J Symes, Geoffrey C.S. Smith

**Affiliations:** 1 Faculty of Medicine, University of New South Wales, Sydney, AUS; 2 Department of Orthopaedic Surgery, St George and Sutherland Hospitals, Sydney, AUS; 3 Department of Orthopaedic Surgery, Royal North Shore Hospital, Sydney, AUS; 4 SCORe, St George and Sutherland Centre for Orthopaedic Research, Sydney, AUS

**Keywords:** career satisfaction, generation, medical, mental toughness, physical activity, resilience, specialty, survey

## Abstract

Background

How mental and physical attributes differ between doctors of different generations and specialities is undetermined. We aimed to compare how resilience, mental toughness, physical activity levels, and career satisfaction vary between medical professionals of different generations, specialities, career stages, practice settings, and genders.

Methodology

This was an electronic survey study that compared the Brief Resilience Score (maximum six points), Mental Toughness Index (maximum 56 points), International Physical Activity Questionnaire-Short Form, and career satisfaction (0-100 scale) between doctors and medical students at two affiliated teaching hospitals. All data are presented as median (interquartile range).

Results

In total, 289 medics responded. Baby Boomers (born 1946-1964) (4.2 (3.7, 4.8)) were more resilient than Generation Z (born 1997-2012) (3.8 (3.2, 4.0), p=0.0007), Millennials (born 1981-1996) (3.8 (3.3, 4.2), p=0.0091), and Generation X (born 1965-1980) (4.0 (3.5, 4.2), p = 0.0354). Generation Z (42 (39, 46)) were less mentally tough than Millennials (44 (41, 47), p=0.0330), Generation X (46 (41, 48), p=0.0062), and Baby Boomers (46 (43, 52), p=0.0005). Millennials were less mentally tough than Baby Boomers (p=0.0199). Generation Z were more physically active than Generation X (1,822 (1,052, 3,662) vs. 1,542 (777, 2,586), p=0.0383). Generation Z had lower career satisfaction than Millennials (73 (58, 80) vs. 80 (70, 89), p=0.0094), Generation X (85 (75, 90), p<0.0001), and Baby Boomers (90 (85, 94), p<0.0001). Millennials had lower career satisfaction than Generation X (p=0.0291) and Baby Boomers (p=0.0014), respectively. Medical students were less resilient than consultants(3.7 (3.2, 4.0) vs. 4.0 (3.5, 4.2), p=0.0193). Medical students (41 (38, 47)) and interns (42 (38, 45)) had lower mental toughness than both senior trainees (46.0 (41, 49), p=0.0096 and p=0.0022, respectively) and consultants (46 (41, 48), p=0.0022 and p=0.0005, respectively). Medical students (2,224 (1,370, 4,760) had higher physical activity levels (as defined by the International Physical Activity Questionnaire-Short Form score) than junior trainees (1,386 (731, 2,656), p=0.0038), senior trainees (1,395 (1,065, 3,034), p=0.0327), and consultants (1,533 (820, 2,546), p=0.0017). Career satisfaction increased with career stage (Spearman’s (r=0.3219, 95% CI=0.2075-0.4277, p<0.001). Physician trainees were more satisfied with their careers (as per self-rated 0-100 satisfaction scale) than trainees of “other” (non-physician/non-surgical) specialities (80 (72, 90) vs. 75 (40, 80), p=0.0090). Consultant surgeons (4.2 (4.0, 4.5) and 48 (46, 53)) were more resilient and mentally tougher than consultant physicians (3.8 (3.3, 4.0) and 44 (39, 48), p=0.0002 and p=0.0003, respectively) and consultants of other specialities (3.8 (3.3, 4.2) and 44 (41, 47), p=0.0004 and p<0.0001, respectively). Consultants who worked in both private and public hospitals had greater mental toughness (47 (43, 51) vs. 44 (41, 47), p=0.0153) and career satisfaction (86 (80, 90) vs. 80 (70, 90), p=0.0053) than consultants who worked exclusively in public hospitals.

Conclusions

The present study found that self-reported resilience and mental toughness were the greatest among the Baby Boomers, and declined with subsequent generations. Among consultants, surgeons had the highest resilience and mental toughness scores. Career satisfaction increased with career stage.

## Introduction

Resilience is defined as a person’s ability to recover quickly after something unpleasant [1]. The ability to bounce back from setbacks and improve as a result is important in all walks of life. With up to 50% of physicians suffering from some form of burnout, resilience and mental toughness are of particular importance to those of us in the medical profession [2,3].

Differences in attitudes between generations are undoubtedly shaped by the time period. However, to view subsequent generations as deficient, especially in aspects or areas in which previous generations excelled, is nothing new [4]. Generational differences have and will always exist, although how these differences translate with respect to resilience and mindset is less well-defined.

The diversity in medical specialities and the corresponding variation in scope of practice, competitiveness, and lifestyle means that there is a speciality for every personality. The privilege of providing lifesaving and/or quality-of-life-improving care requires an often sustained track record of academic excellence, and the journey from medical student to fully qualified specialist is a long and multidimensionally costly endeavor [5,6]. Among the medical profession, surgical training is notoriously competitive and arduous, which suggests that values such as resilience and mental toughness would be particularly evident in this cohort.

Previous satirical investigations have, in ways, validated this stereotype, showing that surgeons were objectively better looking and taller than their physician counterparts, and that orthopedic surgeons in particular were more intelligent and had greater grip strength than their anesthetic colleagues [7,8]. Numerous studies have also shown a positive relationship between physical activity levels and mental attributes such as resilience, mental toughness, and grit [9-14].

The aim of the present study, therefore, was to compare how resilience, mental toughness, physical activity levels, and career satisfaction vary between medical practitioners of different generations, career stages, specialities, practice settings, and genders.

## Materials and methods

Study design

This was a survey of medical practitioners and medical students from St George Hospital and the Sutherland Hospital, two affiliated teaching hospitals of the University of New South Wales, located in the South Eastern Sydney Local Health District (NSW, Australia), where there are approximately 600 doctors, many of whom are dual-employed at both sites. St George Hospital is a 550-bed Level-1 trauma center and tertiary referral hospital for the 250,000 residents of Southern Sydney. The Sutherland Hospital is a 375-bed peripheral hospital of St George Hospital. Respondents were invited to participate via email, WhatsApp, newsletters, posters, and were also appealed to in-person at grand rounds and department meetings. Study data were collected and managed using REDCap electronic data capture tools hosted at the University of New South Wales [15,16].

Inclusion and exclusion criteria

Registered medical practitioners and medical students based at St George Hospital and/or the Sutherland Hospital were eligible for inclusion in the survey. Due to the challenge of obtaining a representative number of responses from staff who did not fall into the category of either medical practitioner or medical student, these individuals were excluded from the study.

Ethical approval

This study was undertaken following the acquisition of ethical approval from the South Eastern Sydney Local Health District Human Research Ethics Committee (protocol reference number: PID/202301594, application number: 2023/ETH01398).

Survey contents

Demographic variables collected were birth year, gender (female, male, other), stage of career (medical student, intern/resident (postgraduate year (PGY) 1-2), junior registrar/basic physician trainee (PGY 3-5), senior registrar/advanced trainee (PGY ≥6), fellow (post-training), consultant), and speciality (for medical students, interns/residents, their speciality of interest). Consultants and fellows were asked whether they worked exclusively in the public hospital system or worked in both the public and private systems.

Participants were asked: how satisfied are you with your career (0-100 visual analogue scale), and three validated questionnaires: Brief Resilience Scale, Mental Toughness Index, and the International Physical Activity Questionnaire-Short Form [17-19]. The survey was designed to take approximately three minutes to complete.

The Brief Resilience Scale is a six-item questionnaire. Participants grade their response to each question using a five-point Likert scale, and the questionnaire is scored as the average of the six items (higher score = greater resilience, maximum score = six points). The Mental Toughness Index is an eight-item questionnaire. Participants grade their response to each question using a seven-point Likert scale, and the questionnaire is scored as the sum of the eight items (higher score = greater mental toughness, maximum score = 56 points). The International Physical Activity Questionnaire-Short Form is an eight-item questionnaire that queries participants on their weekly volume of physical activity, and is reported in MET minutes. METs are multiples of the resting metabolic rate (walking = 3.3 METs, moderate physical activity = 4 METs, and vigorous physical activity = 8 METs), and a MET minute is calculated by multiplying the total number of METs for each activity by the number of minutes spent performing each activity, by the number of days spent each week performing each respective level of activity.

Definitions

Participants born between 1997 and 2012 were classified as Generation Z, those born between 1981 and 1996 were classified as Millennials, those born between 1965 and 1980 were classified as Generation X, and those born between 1946 and 1964 were classified as Baby Boomers. A trainee was defined as someone with three or more postgraduate years (PGY ≥3) of experience as a doctor, but who is not yet a fully qualified/credentialed specialist. A fellow was defined as a recently qualified/credentialed specialist undergoing further sub-specialist training. A consultant was defined as a fully qualified/credentialed practicing specialist. Participants who are in the process of completing or who have completed formal specialist training programs administered by the Royal Australasian College of Surgeons were classified as surgeons. Those who have completed or who are completing specialist training programs administered by the Royal Australasian College of Physicians were classified as physicians. Those whose specialities were not covered by either of the two aforementioned colleges were classified as “other.”

Statistical analysis

Responses were summarized quantitatively or, where appropriate, with descriptive statistics. The skewness of continuous variables was assessed using quantile-quantile (Q-Q) plots. As all continuous variables were skewed, the data were presented as median (interquartile range (IQR)). Nonparametric continuous variables were compared using Mann-Whitney U tests. Significance was set at p-values <0.05. Statistical analysis was conducted using GraphPad Prism (Ver 8.4.2, GraphPad Software Inc, La Jolla, CA, USA).

## Results

Respondent characteristics

There were a total of 289 respondents to the survey. Of the 280 participants who disclosed their date of birth, 90 (32%) were from Generation Z (1997-2012), 106 (38%) were Millennials (1981-1996), 59 (21%) were from Generation X (1965-1980), and 25 (9%) were Baby Boomers (1946-1964). The median (IQR) age was 35 (27, 47) years. Of the 286 participants who disclosed their gender, 152 (53%) were males.

Of the 283 participants who disclosed their career stage, 40 (14%) were medical students, 45 (16%) were interns/residents (postgraduate year (PGY) 1-2), 34 (12%) were junior trainees (PGY 3-5), 40 (14%) were senior trainees (PGY ≥6), 3 (1%) were fellows (post-training), and 122 (43%) were consultants.

Of the 74 trainees (PGY ≥3) who disclosed their speciality, 21 (28%) were surgical trainees, 22 (30%) were physician trainees, and 31 (42%) were trainees from other specialities. Of the 123 consultants who disclosed their speciality and practice types, 27 (22%) were surgeons, 48 (39%) were physicians, and 48 (39%) were consultants from 'other' (non-surgical and non-physician) specialities. Overall, 68 (55%) consultants worked in both the public and private hospital systems, whereas the other 55 (45%) consultants worked exclusively in the public hospital system. Data are presented as median (IQR).

Generation

**Figure 1:**
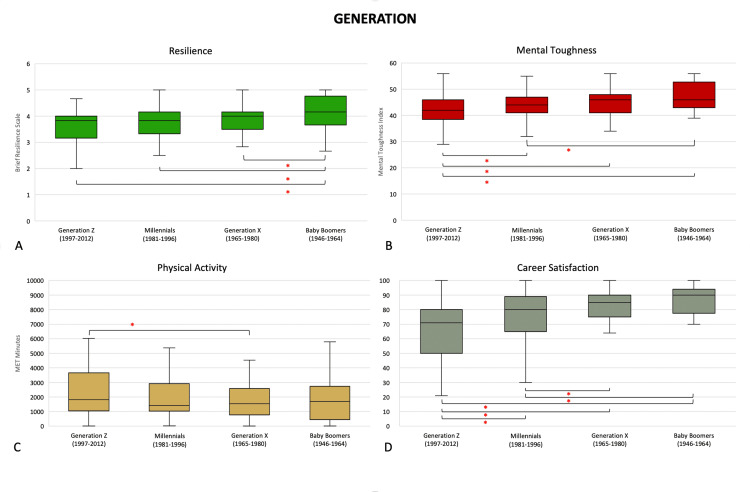
Differences in (A) resilience, (B) mental toughness, (C) physical activity levels, and (D) career satisfaction by generation. *: p <0.05 (Mann-Whitney U test). Middle represents median; upper and lower hinges represent 25% and 75% quartiles; upper and lower whiskers represent 1.5 times and -1.5 times interquartile range.

Baby boomers (4.2 (3.7, 4.8)) were more resilient than Generation Z (3.8 (3.2, 4.0), p = 0.0007), Millennials (3.8 (3.3, 4.2), p = 0.0091), and Generation X (4.0 (3.5, 4.2), p = 0.0354). Generation Z (42 (39, 46)) were less mentally tough than Millennials (44 (41, 47), p = 0.0330), Generation X (46 (41, 48), p = 0.0062), and Baby Boomers (46 (43, 52), p = 0.0005). Millennials were also less mentally tough than Baby Boomers (p = 0.0199). Generation Z (1,822 (1,052-3,662)) was more physically active than Generation X (1,542 (777, 2,586), p = 0.0383). Generation Z (73 (58, 80) had less career satisfaction than Millennials (80 (70, 89), p = 0.0094), Generation X (85 (75, 90), p < 0.0001) and Baby Boomers (90 (85, 94), p < 0.0001). Millennials were also less satisfied with their careers than both Generation X (p = 0.0291) and the Baby Boomers (p = 0.0014) (Figure 1).

**Figure 2:**
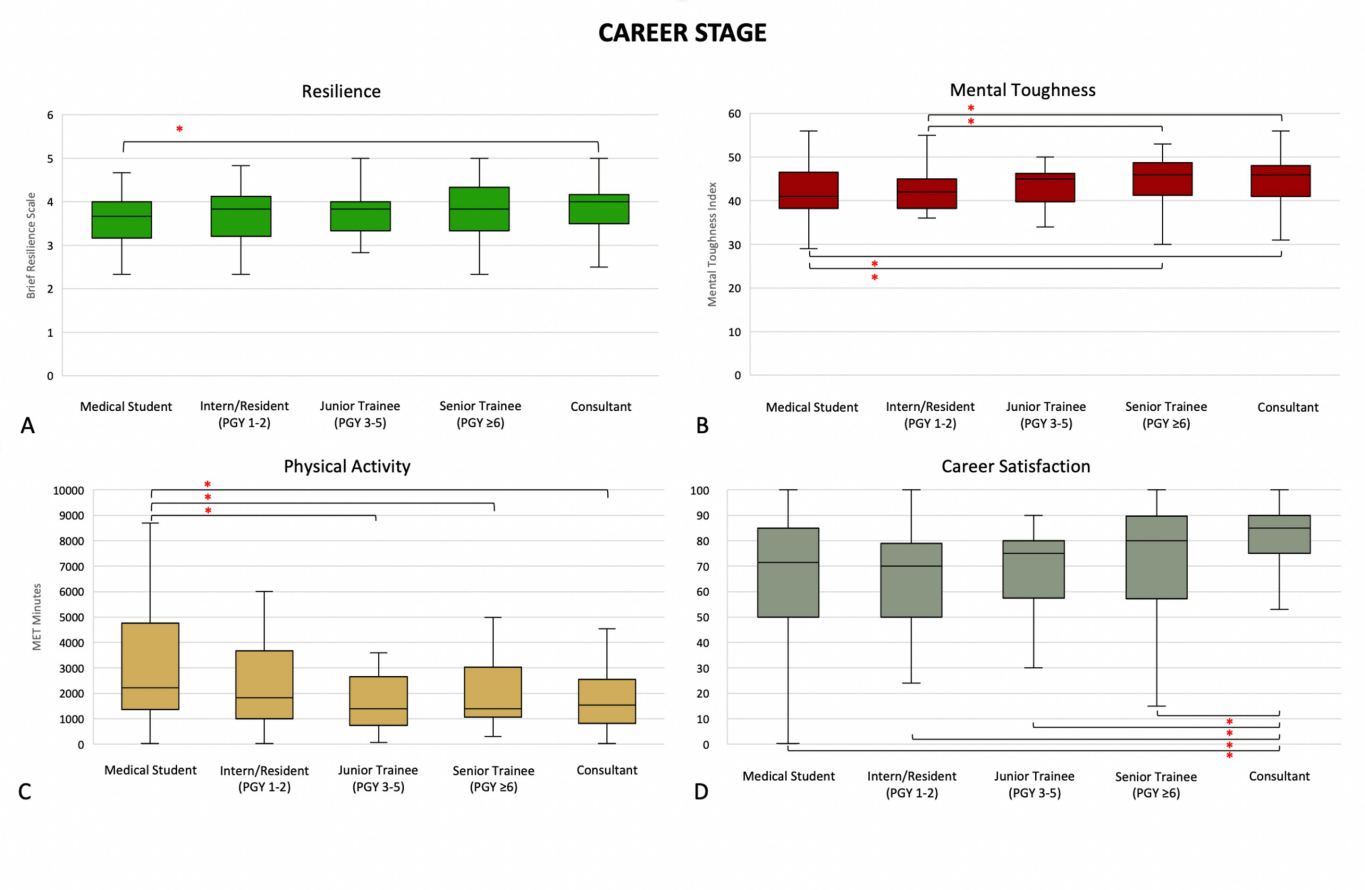
Differences in (A) resilience, (B) mental toughness, (C) physical activity levels, and (D) career satisfaction by career stage. *: p <0.05 (Mann-Whitney U test). Middle represents median; upper and lower hinges represent 25% and 75% quartiles; upper and lower whiskers represent 1.5 times and -1.5 times interquartile range.

Medical students (3.7 (3.2-4.0)) were less resilient than consultants (4.0 (3.5, 4.2), p = 0.0193). Medical students (41 (38, 47)) and interns (42 (38, 45)) had less mental toughness than both senior trainees (46.0 (41, 49), p = 0.0096 and p = 0.0022, respectively) and consultants (46 (41, 48), p = 0.0022 and p = 0.0005, respectively). Medical students (2,224 (1,370, 4,760) had higher physical activity levels than junior trainees (1,386 (731, 2,656), p = 0.0038), senior trainees (1,395 (1,065, 3,034), p = 0.0327), and consultants (1,533 (820, 2,546), p = 0.0017). Consultants had significantly greater career satisfaction than medical students (73 (53, 83), p = 0.0004), interns (73 (57, 80), p < 0.0001), junior trainees (79 (68, 80), p = 0.0003), and senior trainees (80 (70, 90), p = 0.0171) (Figure 2).

**Figure 3:**
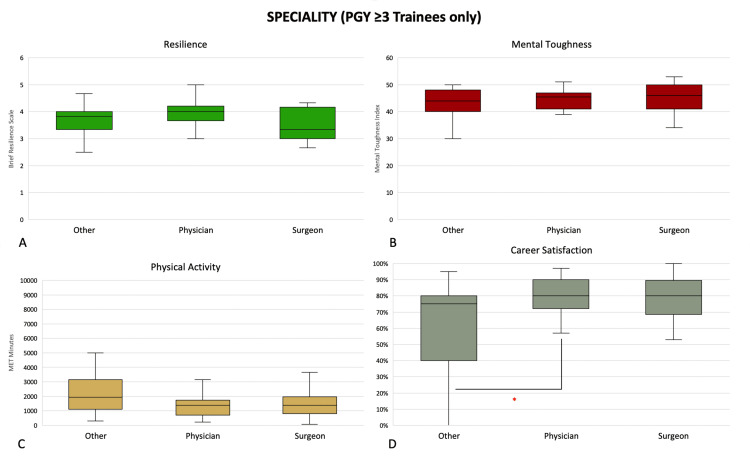
Differences in (A) resilience, (B) mental toughness, (C) physical activity levels, and (D) career satisfaction in trainees (PGY ≥3) grouped by speciality. *: p <0.05 (Mann-Whitney U test). Middle represents median; upper and lower hinges represent 25% and 75% quartiles; upper and lower whiskers represent 1.5 times and -1.5 times interquartile range. PGY = postgraduate year

**Figure 4:**
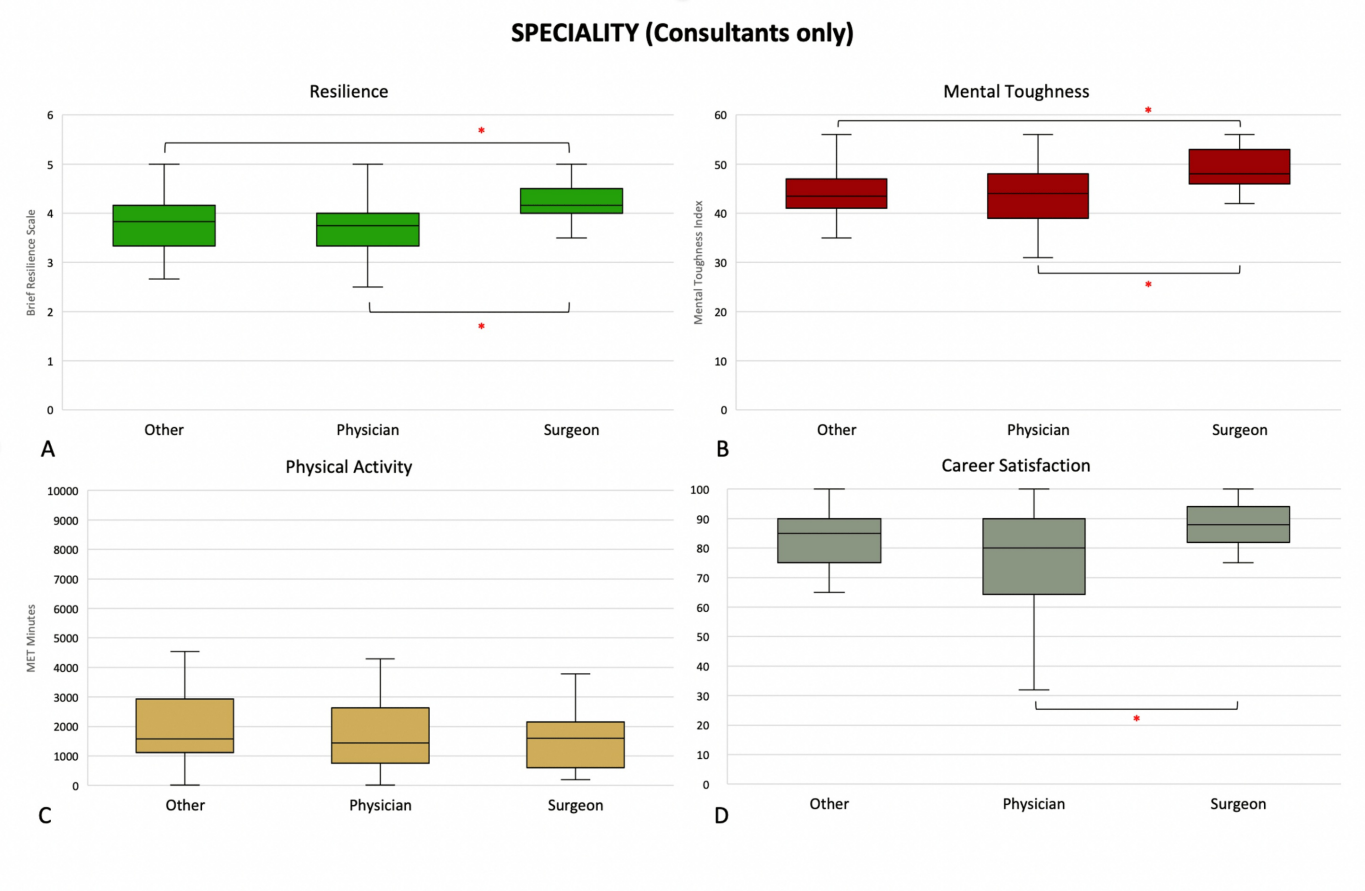
Differences in (A) resilience, (B) mental toughness, (C) physical activity levels, and (D) career satisfaction in consultants grouped by speciality. *: p <0.05 (Mann-Whitney U test). Middle represents median; upper and lower hinges represent 25% and 75% quartiles; upper and lower whiskers represent 1.5 times and -1.5 times interquartile range.

Among trainees (PGY ≥3), those engaged in physician training were significantly more satisfied with their careers than trainees of other non-physician and non-surgical specialities (80 (72, 90) vs. 75 (50, 80), p = 0.0090) (Figure 3). Among consultants, surgeons (4.17 (4.00, 4.50) and 48.0 (46.0, 53.0)) were more resilient and mentally tougher than physicians (3.75 (3.33, 4.00) and 44.0 (39.0, 48.0), p = 0.0002 and p = 0.0003, respectively) and consultants of other specialities (3.83 (3.33, 4.17) and 43.5 (41.0, 47.0), p = 0.0004 and p < 0.0001, respectively) (Figure 4).

**Figure 5:**
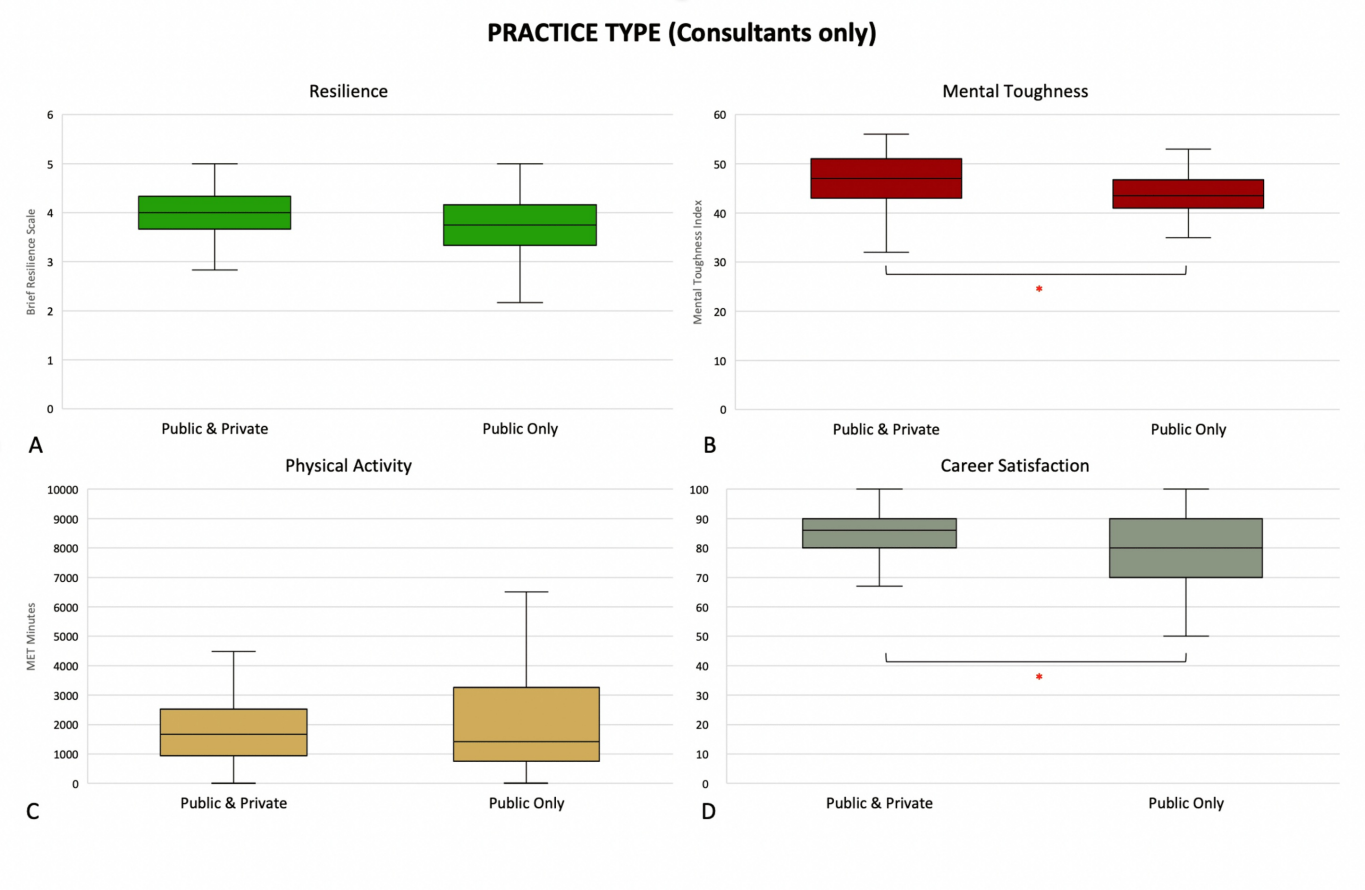
Differences in (A) resilience, (B) mental toughness, (C) physical activity levels, and (D) career satisfaction in consultants grouped by practice type. *: p <0.05 (Mann-Whitney U test). Middle represents median; upper and lower hinges represent 25% and 75% quartiles; upper and lower whiskers represent 1.5 times and -1.5 times interquartile range.

**Figure 6:**
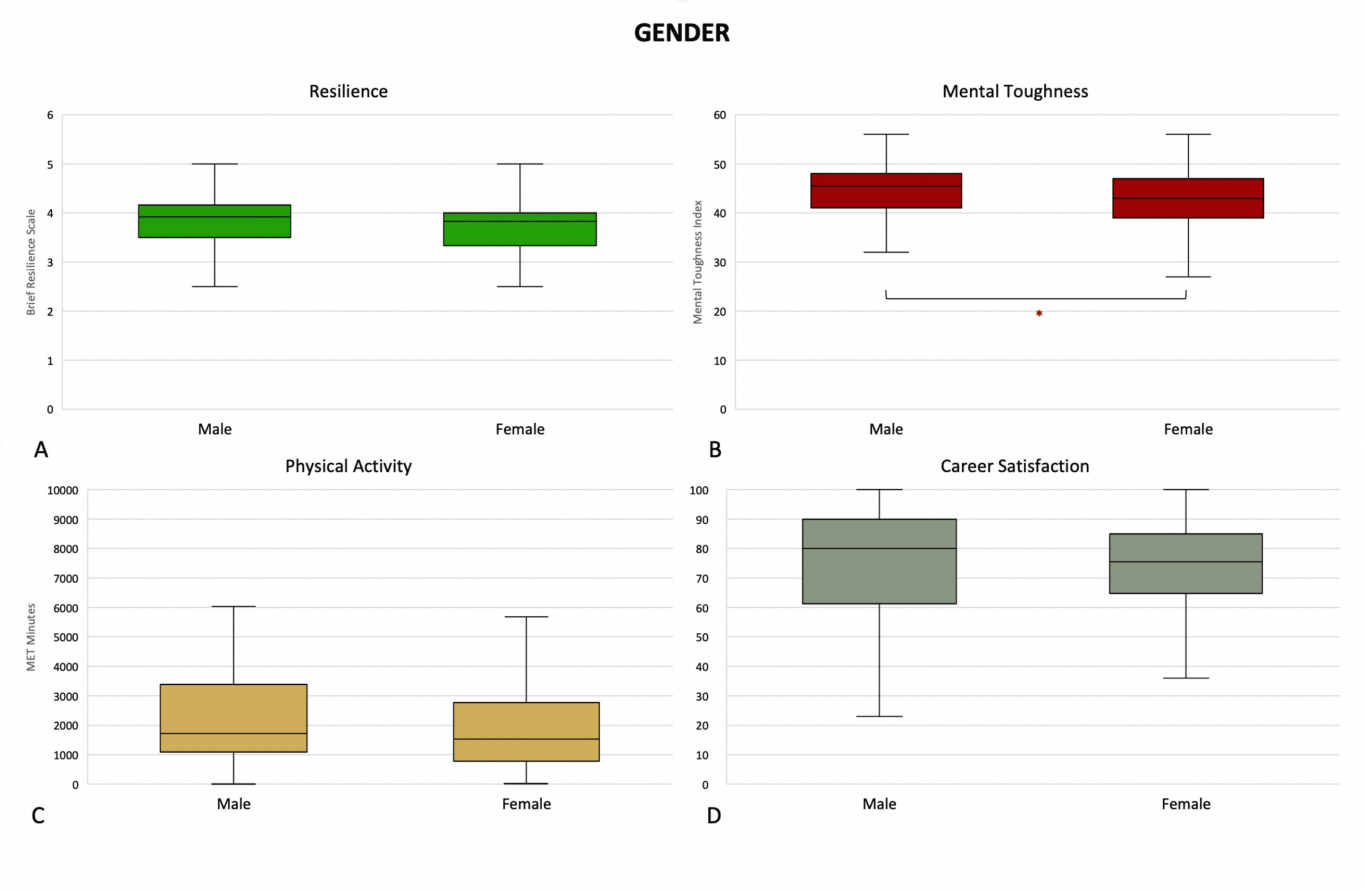
Differences in (A) resilience, (B) mental toughness, (C) physical activity levels, and (D) career satisfaction by gender. *: p <0.05 (Mann-Whitney U test). Middle represents median; upper and lower hinges represent 25% and 75% quartiles; upper and lower whiskers represent 1.5 times and -1.5 times interquartile range.

Consultants engaged in both private and public work had greater mental toughness (47 (43, 51) vs. 44 (41, 47), p = 0.0153) and career satisfaction (86 (80, 90) vs. 80 (70, 90), p = 0.0053) than their colleagues who worked exclusively in the public hospital system (Figure 5). Male respondents had greater self-reported mental toughness (46 (41, 48) vs. 43 (39, 47), p = 0.0173) than female respondents (Figure 6).

## Discussion

The major finding of this study was that the Baby Boomer generation and surgeons were more resilient and mentally tougher than those of other generations and specialities respectively. Resilience, mental toughness, and career satisfaction increased linearly with age and career stage. Younger cohorts’ mental attributes were not bolstered by greater physical activity levels. While career satisfaction typically increases with progression, lower resilience and toughness in younger generations may reflect societal changes. In Australia, medical working conditions have improved significantly, with an emphasis on mental health and wellbeing [20,21]. Perhaps the new generation is more introspective and/or self-critical. While resilience, like so many other critical attributes, may be developed over time, this may also reflect changes in social, economic, and environmental factors [22].

Physician trainees were significantly more satisfied with their careers than trainees of “other” specialities. Consultant-wise, surgeons were more resilient and mentally tougher than both physicians and “other” specialists, with no difference in activity levels. While some might argue that mentality differences may be explained by the rigor of completing surgical training in the previous era, others may point to the finding that surgeons were the most likely doctors to demonstrate narcissistic personality traits [23].

Consultant surgeons were also significantly more satisfied with their careers than consultants of “other” specialities. In contrast, a study of 8,108 American doctors found that while no surgical speciality was significantly more likely to be “very satisfying” than general practice, three of the five specialities that were significantly less likely to be “very satisfying” than general practice were surgical specialities [24].

Interestingly, consultants who worked in both public and private hospitals had greater mental toughness scores and were more satisfied with their careers than those who worked exclusively in public hospitals. This difference in mental toughness may extend beyond clinical workload, potentially reflecting financial challenges in private practice early in one’s career [25,26]. However, it would seem that, ultimately, engaging in both private and public practice is a rewarding endeavor.

The finding that male respondents had higher self-rated mental toughness than female respondents is likely because males are significantly more likely to overestimate their own abilities than females, without a difference in competence [27,28]. Dunning et al. surveyed college students about their self-perceived scientific knowledge, and subsequently invited them to complete a quiz. They found that while females were less confident about their knowledge than males in the pre-quiz survey, there was no difference in scores between groups [27]. Interestingly, when queried about their performance post-quiz, although both genders underestimated their performance, females did so to a greater degree, a phenomenon that likely occurred in the present study.

This study had several limitations. This was an observational study that was limited to two affiliated teaching hospitals within a single health district in Australia; therefore, these findings cannot be extrapolated to all Australian doctors or international settings. (i.e., age-career stage overlap, multiple testing). Furthermore, given that the primary aim of this study was to screen for differences between generational/career stage/specialty/demographic groups using validated outcome measures, rather than to model causal relationships, the study was not designed or powered to adjust for potential confounders related to age or career stage. Accordingly, findings should be interpreted in the context of unadjusted between-group comparisons. As with all survey-based studies, there was the potential for selection bias, response bias, survey fatigue, and recall bias. With particular regard to the Brief Resilience Scale and Mental Toughness Index, some might argue that these were more a measure of hubris rather than of resilience or mental toughness. A strength of this study was the use of well-validated grading systems for our main outcomes.

## Conclusions

The Baby Boomers were significantly more resilient and mentally tougher than respondents of subsequent generations. Career satisfaction increased linearly with career stage. Among consultants, surgeons had the highest self-reported resilience and mental toughness scores. However, these mental attributes were not maintained when comparing trainees based on speciality.
